# Different Natural Selection Pressures on the *atpF* Gene in Evergreen Sclerophyllous and Deciduous Oak Species: Evidence from Comparative Analysis of the Complete Chloroplast Genome of *Quercus aquifolioides* with Other Oak Species

**DOI:** 10.3390/ijms19041042

**Published:** 2018-03-30

**Authors:** Kangquan Yin, Yue Zhang, Yuejuan Li, Fang K. Du

**Affiliations:** College of Forestry, Beijing Forestry University, Beijing 100083, China; yinkq@im.ac.cn (K.Y.); zhangyue2016@bjfu.edu.cn (Y.Z.); liyuejuan@bjfu.edu.cn (Y.L.)

**Keywords:** cp genome, repeat analysis, sequence divergence, non-synonymous substitution, electron transport chain, phylogeny

## Abstract

*Quercus* is an economically important and phylogenetically complex genus in the family Fagaceae. Due to extensive hybridization and introgression, it is considered to be one of the most challenging plant taxa, both taxonomically and phylogenetically. *Quercus aquifolioides* is an evergreen sclerophyllous oak species that is endemic to, but widely distributed across, the Hengduanshan Biodiversity Hotspot in the Eastern Himalayas. Here, we compared the fully assembled chloroplast (cp) genome of *Q. aquifolioides* with those of three closely related species. The analysis revealed a cp genome ranging in size from 160,415 to 161,304 bp and with a typical quadripartite structure, composed of two inverted repeats (IRs) separated by a small single copy (SSC) and a large single copy (LSC) region. The genome organization, gene number, gene order, and GC content of these four *Quercus* cp genomes are similar to those of many angiosperm cp genomes. We also analyzed the *Q. aquifolioides* repeats and microsatellites. Investigating the effects of selection events on shared protein-coding genes using the Ka/Ks ratio showed that significant positive selection had acted on the *atpF* gene of *Q. aquifolioides* compared to two deciduous oak species, and that there had been significant purifying selection on the *atpF* gene in the chloroplast of evergreen sclerophyllous oak trees. In addition, site-specific selection analysis identified positively selected sites in 12 genes. Phylogenetic analysis based on shared protein-coding genes from 14 species defined *Q. aquifolioides* as belonging to sect. *Heterobalanus* and being closely related to *Q. rubra* and *Q. aliena*. Our findings provide valuable genetic information for use in accurately identifying species, resolving taxonomy, and reconstructing the phylogeny of the genus *Quercus*.

## 1. Introduction

The chloroplast (cp) is an organelle which plays an important role in photosynthesis and carbon fixation in plant cells. In angiosperms, the cp is a uniparentally inherited organelle, and it has its own circular, haploid, evolutionarily conserved genome. The cp genome is therefore considered to be a useful and informative genetic resource for studies on evolutionary relationships in the plant kingdom at various taxonomic levels [1]. In most cases, the cp genome is between 120 and 160 kb in size and has a structure composed of two copies of a large inverted repeat (IR) region, a large single copy (LSC) region, and a small single copy (SSC) region [2]. 

Oaks (*Quercus* L.), which comprise approximately 500 shrub and tree species, form a phylogenetically complex and economically important genus of the beech family, Fagaceae [3]. Distributed throughout much of the Northern Hemisphere, oaks are located in the northern temperate region, and they also occur in the Andes of South America and subtropical and tropical Asia [4]. Oaks are dominant in various habitats, such as temperate deciduous forest, oak-pine forest and temperate and subtropical evergreen forest [5]. They are intimately associated with many other organisms, including fungi, ferns, birds, mammals, and insects [4]. For this reason, their interactions have been the subject of a large number of ecological studies. Human beings have a close connection with oak, as throughout history it has been a common symbol of strength and courage and has been chosen as the national tree in many countries. Moreover, oaks are of great economic value, being used in, for example, the construction of fine furniture and the wine industry. 

Oak species are notoriously difficult to classify taxonomically, due to morphological variation caused in part by hybridization [6,7,8,9,10,11,12,13,14]. Some studies stated that *Quercus* contained two subgenera, *Cyclobalanopsis* and *Quercus*, the latter including three sections: *Quercus* (white oaks), *Lobatae* (red oaks), and *Protobalanus* (golden cup or intermediate oaks) [3,15]. Because previous classifications of oaks have been based solely on morphological characters which are often homoplastic in oaks, these classifications have always been subject to debate [3,15]. With advances in molecular phylogenetics and techniques based on pollen morphology, views on oak classification are changing [15,16,17,18,19]. Recently, Denk et al. proposed an updated classification for *Quercus* with two subgenera: subgenus *Quercus*, the ‘New World clade’ or ‘high-latitude clade’, and subgenus *Cerris*, the exclusively Eurasian ‘Old World clade’ or ‘mid-latitude clade’ [19]. There are five sections (*Protobalanus*, *Ponticae*, *Virentes*, *Quercus*, and *Lobatae*) in subgenus *Quercus* and three sections (*Cyclobalanopsis*, *Ilex* and *Cerris*) in subgenus *Cerris*. 

China, which is a center of *Quercus* diversity, has 35–51 species [20]. Based on morphological characters, including 25 qualitative and 18 quantitative characters, oaks in China were divided into five sections, namely *Aegilops*, *Quercus*, *Brachylepides*, *Engleriana*, and *Echinolepides*. Recently, we studied the phylogeography of *Quercus aquifolioides*, which is endemic to the Hengduanshan Biodiversity Hotspot, based on 58 populations distributed throughout the species range, using four chloroplast DNA fragments and 11 nuclear microsatellite loci [21]. Up till now, to our knowledge, very few studies have focused on the phylogenetic relationships and population genetics of oaks in China [22], in part due to the challenges arising from introgressive hybridization, lineage sorting, and molecular markers failing to give sufficient phylogenetic signals. 

In this study, we produced the first cp genome sequence for *Q. aquifolioides* using next-generation sequencing technology. This complete cp genome, combined with previously reported cp genome sequences for other members of the genus, will enhance our understanding of the systematic evolution of *Quercus*. We analyzed the completely assembled cp genome of *Q. aquifolioides* and compared it to those of three other oak species to investigate common structural patterns and hotspot regions of sequence divergence in these four *Quercus* cp genomes, examined whether selection pressure had acted on protein coding genes, and reconstructed the phylogenetic relationships of the four *Quercus* species. Our findings will not only enrich the complete cp genome resources available for the genus *Quercus* but also provide abundant genetic information for use in subsequent taxonomic and phylogenetic identification of members of the genus, and assist geneticists and breeders in improving commercially-grown oak trees.

## 2. Results and Discussion

### 2.1. Chloroplast Genome Organization in Q. aquifolioides

**Figure 1:**
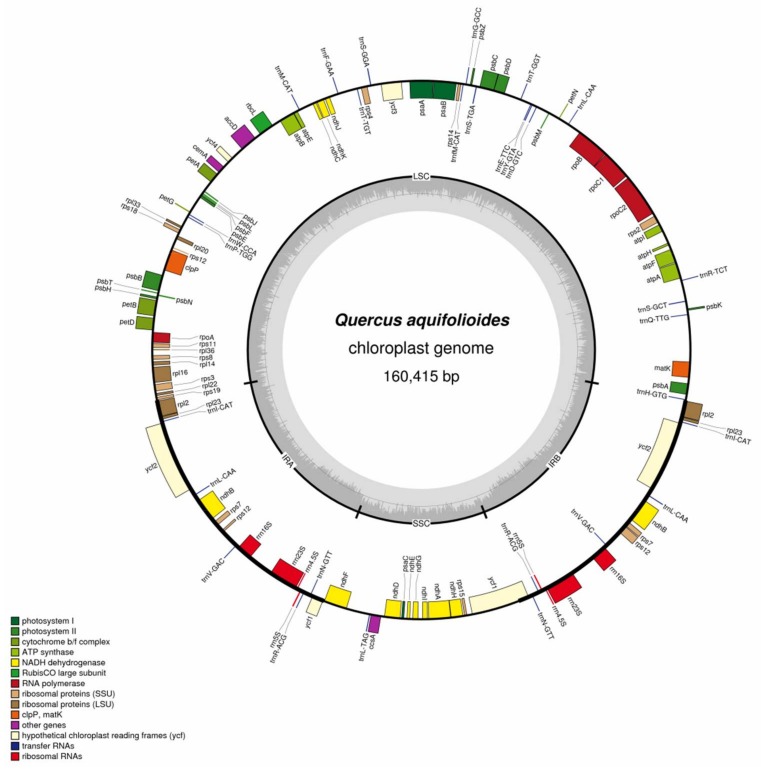
Gene map of the *Q. aquifolioides* chloroplast genome. The annotated chloroplast (cp) genome of *Q. aquifolioides* is represented as concentric circles. Genes shown outside the outer circle are transcribed counter-clockwise and genes indicated inside the outer circle are transcribed clockwise. Two inverted repeats (IRs), the large single copy (LSC) and the small single copy (SSC) are shown in the inner circle.

The *Q. aquifolioides* cp genome is a typical circular double-stranded DNA molecule with a length of 160,415 bp, which falls within the normal angiosperm length range [23,24]. The cp genome has the usual quadripartite structure, featuring a LSC region (large single copy region, 89,493 bp), a SSC region (small single copy region, 16,594 bp), and a pair of IRs (inverted repeats, 25,857 bp) (Figure 1; GenBank accession No. KP340971). The GC contents of the LSC, SSC, and IR regions individually, and of the cp genome as a whole, are 34.8%, 31.2%, 42.7%, and 36.9%, respectively. These GC contents are within the range previously reported for other plant species. Approximately 48.0% of the cp genome encodes proteins, 5.6% encodes rRNAs and 1.3% encodes tRNAs. Noncoding regions (intergenic regions, introns and pseudogenes) constitute the remaining 45.1% of the genome. The *Q. aquifolioides* cp genome encodes 127 genes: 80 protein-coding genes, eight ribosomal RNA genes, and 39 tRNA genes. *ycf2* is the largest gene, having a length of 6834 bp. We found that 18 genes have one intron (10 protein coding genes and 8 tRNA genes) and two genes (*clpP* and *ycf3*) have two introns each. Two identical rRNA gene clusters (16S-23S-4.5S-5S) were found in the IR regions. There are two tRNA genes, *trnI* and *trnA*, in the 16S~23S spacer region of each cluster. The sequence of the rRNA coding region is highly conserved: sequence identities of four rRNA genes with those of *Arabidopsis thaliana* (L.) Heynh were over 98%. 

### 2.2. Repeat Sequence Analysis and Simple Sequence Repeats (SSR)

**Figure 2:**
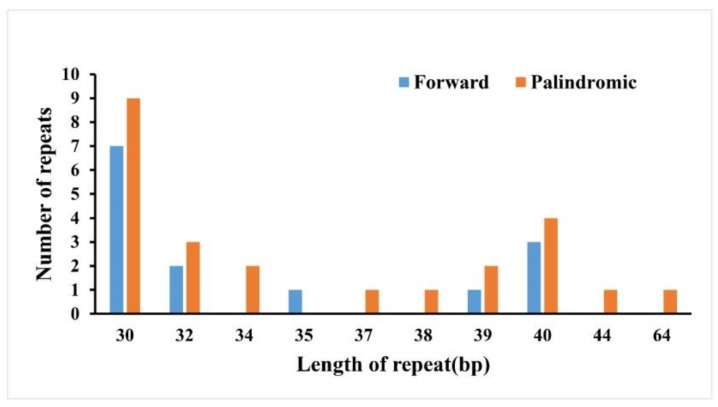
Analysis of repeated sequences in *Q. aquifolioides*.

Repeat sequences have been used extensively for phylogeny, population genetics, genetic mapping, and forensic studies [25]. In the cp genome of *Q. aquifolioides*, 38 pairs of repeats longer than 30 bp were detected; they consisted of 24 palindromic repeats and 14 forward repeats (Figure 2). Among these repeats, 36 are 30–40 bp long, one is 44 bp long, and one is 64 bp long (Figure 2). A large proportion of the repeats (73.7%) are present in non-coding regions, but some repeats are embedded in coding regions, such as the *trnS*-*GCU*, *trnS*-*GGA*, *psaB*, *psaA*, *ycf1*, *ycf2*, and *accD* genes (Table S1). As previous studies reported, many repeats were found in the *ycf2* gene [26,27,28,29]. Apart from the IR region, the longest repeats, which were 64 bp in length, were present in the *ndhD*/*psaC* intergenic region.

**Figure 3:**
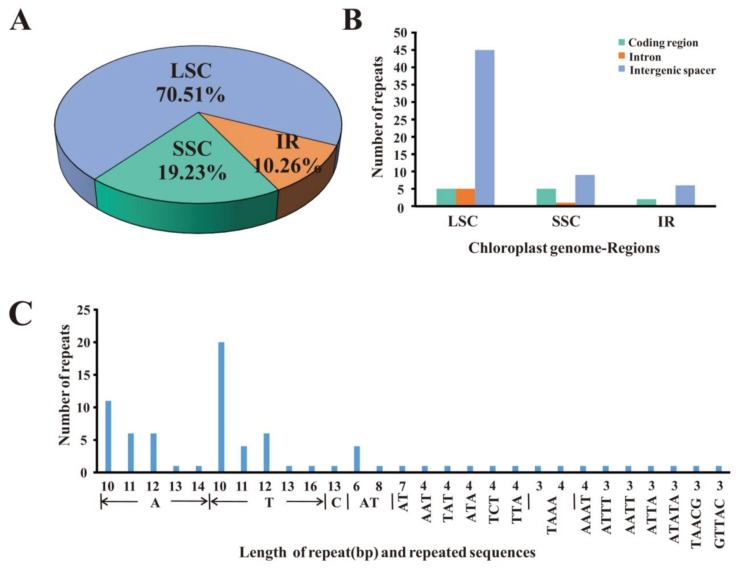
Analysis of simple sequence repeats (SSRs) in the *Q. aquifolioides* cp genome. (**A**) Frequency of SSRs identified in the LSC, SSC, and IR regions; (**B**) Frequency of SSRs identified in the coding regions, intergenic spacers and introns of the LSC, SSC and IR regions; (**C**) Frequency distribution of different classes of polymer in the cp genome of *Q. aquifolioides*.

SSR, also known as microsatellites, are highly polymorphic and thus widely used as molecular markers. A total of 78 perfect microsatellites were identified in the *Q. aquifolioides* cp genome. Among them, 70.51% were present in the LSC regions, whereas 10.26% and 19.23% were identified in the IR and SSC regions respectively (Figure 3A). This result is consistent with previous reports that SSRs are not evenly distributed in cp genomes [30]. Twelve of the SSRs were present in protein-coding regions, six were in introns, and 60 were located in intergenic spacers of the *Q. aquifolioides* cp genome (Figure 3B). Of the motifs forming these SSRs, 58 are mononucleotides, six are dinucleotides, five are trinucleotides, six are tetranucleotides, and three are pentanucleotides (Figure 3C). Most of the mononucleotides (98.28%) and dinucleotides (100%) are composed of A and T. (Figure 3C). These results are consistent with previous reports that SSRs in cp genomes generally consist of short polyA or polyT repeats [31]. The high AT content of cp SSRs contributes to the AT richness of the *Q. aquifolioides* cp genome, which is similar in this respect to other cp genomes [31]. 

### 2.3. Comparison of the cp Genomes of Q. aquifolioides and Three Related Quercus Species

Three complete cp genomes, those of *Q. aliena* (GenBank accession number: KP301144), *Q. rubra* (GenBank accession number: JX970937), and *Q. spinosa* (GenBank accession number: KM841421), belonging to three different sections within the *Quercus* genus, were selected for comparison with *Q. aquifolioides* (Table 1). *Q. rubra* has the largest cp genome; this is mostly attributable to variations in the lengths of the LSC and SSC regions. The GC content of these four cp genomes is very similar, at ~37%. *Q. aquifolioides* has the same number of protein coding genes and rRNA genes as the other three *Quercus* species. Although *Q. spinosa* has one tRNA fewer than the other three *Quercus* species, the total length of its tRNA genes is greater than that in any of the other three species. We found that *Q. aquifolioides* shared 80 protein-coding genes with the cp genomes of all three of the other *Quercus* species. 

**Figure 4:**
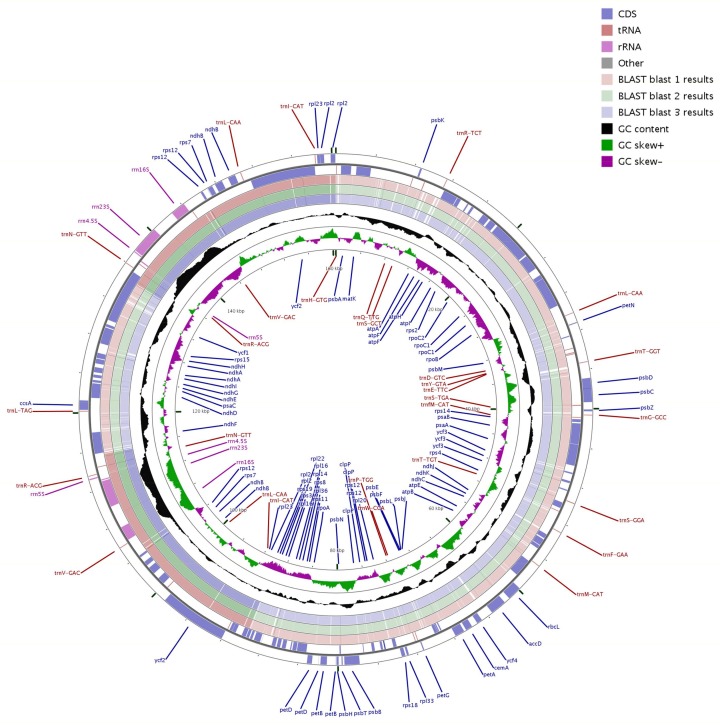
Comparison of four *Quercus* cp genome sequences. The outer four rings show the coding sequences, tRNA genes, rRNA genes, and other genes in the forward and reverse strands. The next three rings show the blast results between the cp genomes of *Q. aquifolioides* and three other *Quercus* species based on BlastN (blast 1–3 results: *Q. aquifolioides* Vs *Q. aliena*, *Q. rubra*, and *Q. spinosa,* respectively). The following black ring is the GC content curve for the *Q. aquifolioides* cp genome. The innermost ring is a GC skew curve for the *Q. aquifolioides* cp genome. GC skew+ (green) indicates G > C, GC skew− (purple) indicates G < C.

We compared the other three complete cp genomes with that of *Q. aquifolioides* (Figure 4). The sequence identity between these four *Quercus* cp genomes was analyzed. Our results revealed perfect conservation of gene order along the cp genomes of the four species and very high similarity between them.

**Figure 5:**
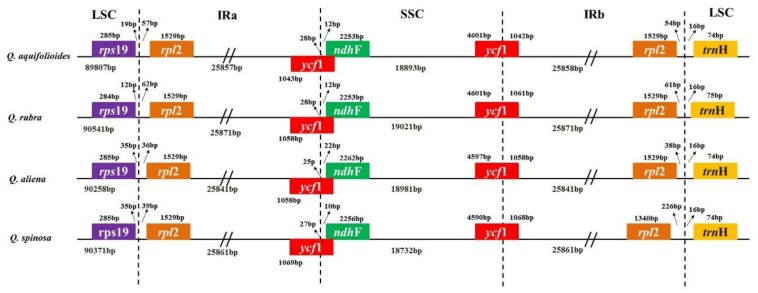
Comparisons of borders between neighboring genes and junctions of the LSC, SSC, and IR regions among the four *Quercus* cp genomes. Boxes above or below the main line indicate genes adjacent to borders. The figure is not to scale with regard to sequence length and shows only relative changes at or near (inverted repeats/single copy) IR/SC borders.

Although the overall quadripartite structure, including the gene number and order, is usually well conserved, the IR region often undergoes expansion or contraction, a phenomenon called ebb and flow in cp genomes [32]. Generally, the expansion or contraction involves no more than a few hundred nucleotides. Kim and Lee proposed that length variation in angiosperm cp genomes was primarily caused by expansion and contraction of the IR region and the single-copy (SC) boundary regions [33]. The IR/SC boundary regions of these four complete *Quercus* cp genomes were compared, and found to exhibit clear differences in junction positions (Figure 5). The inverted repeat b (IRb)/SSC borders are located in the coding region of the *ycf1* gene with a region of 4590–4611 bp located in the SSC regions. The shortened *ycf1* gene crossed the inverted repeat a (IRa)/SSC borders, with 25–28 bp falling within the SSC regions, and the *ndhF* gene was located in the SSC region with its distance to the IRa/SSC borders ranging from 8 to 22 bp. At the LSC/IRa junction, the distances between *rps19* and the border ranged from 12 to 35 bp, while the distances between *rpl2* and the border were from 39 to 63 bp. At the LSC/IRb junction, the distances between *rpl2* and the border ranged from 54 to 226 bp and the distances between *trnH* and the border were the same, at 16 bp. Thus, variations at the IR/SC borders in these four cp genomes contribute to the differences in length of the cp genome sequence as a whole. 

**Figure 6:**
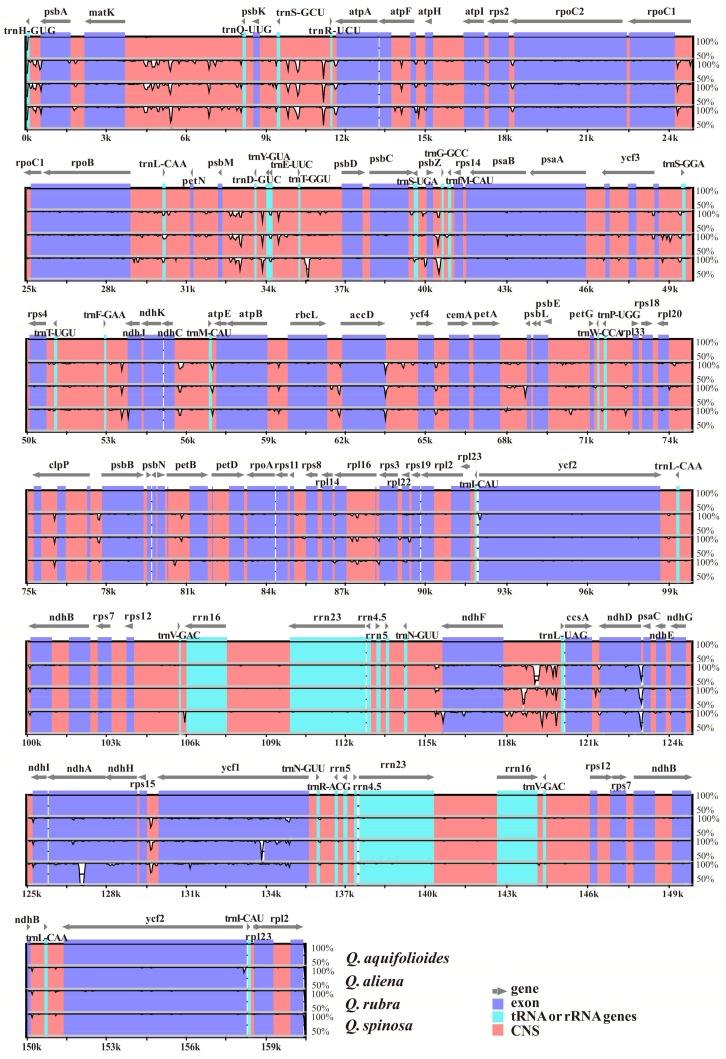
Alignment of four *Quercus* cp genome sequences. Sequence identity plot for four *Quercus* species, with *Q. aquifolioides* as a reference. The *X*-axis corresponds to coordinates within the cp genome. The *Y*-axis shows the percentage identity in the range 50% to 100%.

The whole-genome alignment revealed high sequence similarity across these four cp genomes, suggesting that *Quercus* cp genomes are well conserved (Figure 6). As observed in other angiosperms [34,35,36], we also found that among these four cp genomes the SC regions are more divergent than the IR regions, possibly due to error correction occurring via gene conversion between IRs [37]. Our results also showed that coding regions are more conserved than non-coding regions, as seen in other plants [38,39]. The most divergent coding region in these four *Quercus* cp genomes was *rpl22*. Non-coding regions showed various degrees of sequence divergence among these four *Quercus* cp genomes, with the *trnH-GUG/psbA* regions having the highest level of divergence. These hotspot regions furnish valuable information as a basis for developing molecular markers for phylogenetic studies and identification of *Quercus* species. 

### 2.4. Genome Sequence Divergence among Quercus Species

**Figure 7:**
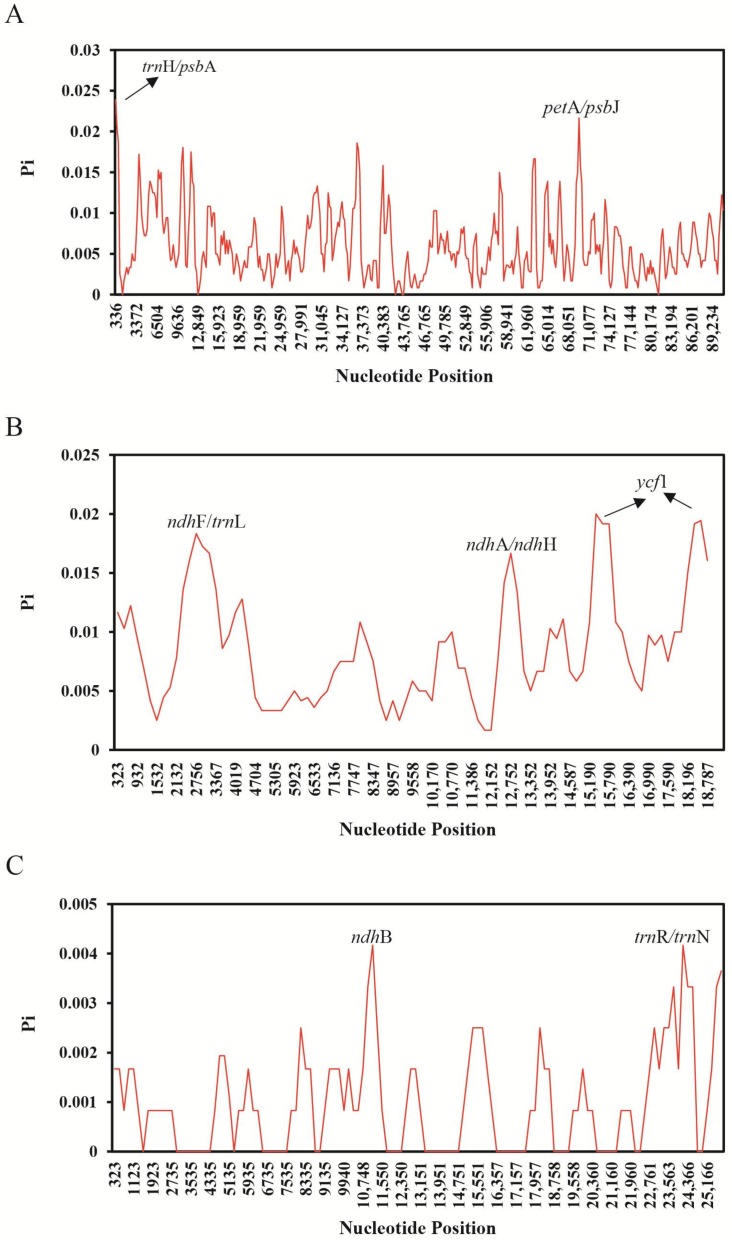
Comparative analysis of nucleotide variability (*Pi*) values among the four *Quercus* cp genome sequences. (**A**) Analysis of the LSC regions; (**B**) Analysis of the SSC regions; (**C**) Analysis of the IR regions. (Window length: 600 bp, step size: 200 bp). *X*-axis: position of the midpoint of a window, *Y*-axis: nucleotide diversity of each window.

**Figure 8:**
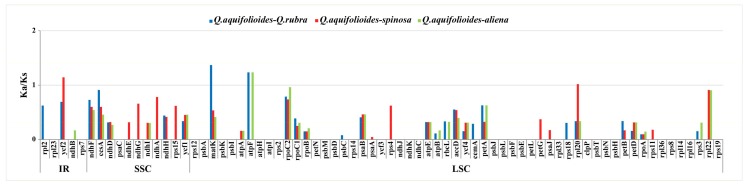
Ka/Ks ratios for protein-coding genes from *Q. rubra*, *Q. spinosa*, and *Q. aliena* chloroplast genome in comparison with *Q. aquifolioides*.

To investigate the extent of sequence divergence among these four *Quercus* cp genomes, the nucleotide variability (*Pi*) values within 600 bp windows (200 bp stepwise moving) in the LSC, SSC, and IR regions of the genomes were calculated (Figure 7). In the LSC region, the values varied from 0 to 0.02389 with a mean of 0.00603, while the SSC regions were from 0 to 0.02 with a mean of 0.00863, and the IR regions were from 0 to 0.00417, with a mean of 0.00098. These results suggest that the differences between these genomic regions are very small. However, we also found certain highly variable regions in the LSC, SSC, and IRs. In the LSC, the highly variable regions were *trnH/psbA* and *petA/psbJ*, with *Pi* > 0.02. In the SSC, highly variable regions included *ndhF/rpl32*, *ndhA/ndhH* and *ycf1* (*Pi* > 0.015). In the IRs, two regions, *trnR/trnN* and *ndhB*, with *Pi* > 0.004 were identified (Figure 8). Four of these regions, *trnH/psbA*, *petA/psbJ*, *ndhF/rpl32*, and *ycf1*, have also been identified as highly variable in other plants [33,40,41,42]. On the basis of our results, five of these variable regions (*trnH/psbA*, *petA/psbJ*, *ndhF/rpl32*, *ndhA/ndhH*, and *ycf1*) show great potential as sources of useful phylogenetic markers for *Quercus*.

### 2.5. Selection Events in Protein Coding Genes

The non-synonymous (Ka) to synonymous (Ks) nucleotide substitution rate ratio (denoted by Ka/Ks) is a very important tool used in studying in protein coding gene evolution. The Ka/Ks ratio is used to evaluate the rate of gene divergence and determine whether positive, purifying or neutral selection has been in operation. A Ka/Ks ratio of >1 indicates positive selection, while Ka/Ks < 1 (especially if it is less than 0.5) indicates purifying selection. A value of close to 1 indicates neutral selection [43].

In this study, we compared Ka/Ks ratios for 73 shared unique protein coding genes in the *Q. aquifolioides* cp genome and the cp genomes of three other related *Quercus* species: *Q. rubra*, *Q. spinosa*, and *Q. aliena* (Table S2). The results are shown in Figure 8. Interestingly, we found that the Ka/Ks ratios were both region specific and gene specific. The average Ka/Ks ratio of the 73 protein coding genes analyzed across the four cp genomes was 0.1653. The most conserved genes with average Ka/Ks values of 0, suggesting very strong purifying selective pressure, were *rpl23*, *rps7*, *psaC*, *rps12*, *psbA*, *psbK*, *psbI*, *atpH*, *atpI*, *rps2*, *petN*, *psbM*, *psbD*, *rps14*, *ycf3*, *ndhJ*, *ndhK*, *ndhC*, *psbJ*, *psbL*, *psbF*, *psbE*, *petL*, *rpl33*, *clpP*, *psbT*, *psbN*, *psbH*, *rpl36*, *rps8*, *rpl14*, *rpl16*, and *rps19* (Table S2). The averaging Ka/Ks method showed no gene with Ka/Ks > 1, which suggests that no gene had been under positive selection in the *Q. aquifolioides* cp genome. Average Ka/Ks value within the range 0.5 to 1, indicating relaxed selection, were observed for *accD*, *petA*, *rpl20*, *rpl22*, *ycf2*, *ndhF*, *ccsA*, *matK*, *atpF*, and *rpoC2*. The remaining genes showed average Ka/Ks values of between 0 and 0.49, which suggested that most genes in the *Q. aquifolioides* cp genome were under purifying selection. 

Although no genes were observed with average Ka/Ks > 1, there were four genes (*ycf2*, *matK*, *atpF*, and *rpl20*) with Ka/Ks > 1 in at least one pairwise comparison (Figure 8). Of these, only *atpF* had Ka/Ks > 1 in two pairwise comparisons. More interestingly, the Ka/Ks ratio for the *atpF* gene was more than 1 in the comparisons with the two deciduous *Quercus* species. In contrast, the Ka/Ks ratio for this gene was 0 in the comparison between *Q. aquifolioides* and *Q. spinosa*, which are both evergreen sclerophyllous oak trees. Deciduous oak trees completely lose their foliage during the winter or the dry season, usually as an adaptation to cold and/or drought, whereas evergreen sclerophyllous oak trees retain their leaves throughout of the year. For leaves to tolerate cold and drought stresses requires energy. The *atpF* gene encodes one of the subunits of the H^+^-ATP synthase, which is essential for electron transport and photophosphorylation during photosynthesis [44]. This finding suggests that differential selection acting on the *atpF* gene may indicate that it has played a role in deciduous-evergreen oak tree divergence.

Alongside the Ka/Ks analysis, we also investigated site-specific selection events. We found a total of 12 genes exhibiting site-specific selection (Table 2). Of these, *rpoC2* was found to have 26 sites under positive selection. This gene encodes one of the four subunits of RNA polymerase type I (plastid-encoded polymerase, PEP), which is a key enzyme required for transcription of photosynthesis-related genes in the chloroplast [45]. Our identification of the positively-selected sites in this analysis could lead to a better understanding of the evolution of *Quercus* species.

### 2.6. Phylogenetic Analysis of the cp Genomes of Q. aquifolioides and Related Quercus Species

**Figure 9:**
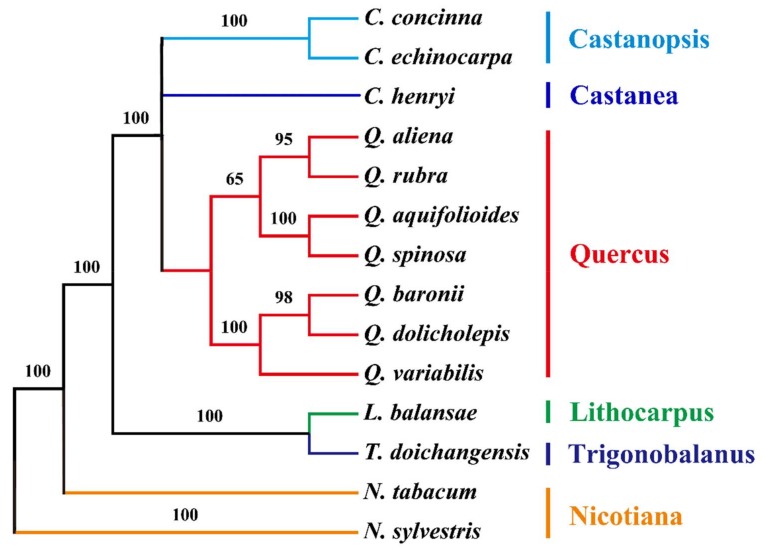
Phylogenetic relationship between *Q. aquifolioides* and related species, inferred from 73 protein-coding genes shared by all cp genomes. The phylogenetic tree was constructed by the maximum parsimony method, with two *Nicotiana* species as outgroups.

The phylogeny of oak trees is complex due to extensive introgression, hybridization, incomplete lineage sorting, and convergent evolution [46]. However, phylogenetic issues in many angiosperms have been addressed successfully with the help of cp genome sequences [47,48,49]. Maximum parsimony (MP) analysis with 73 protein-coding genes from 12 Fragaceae with two tobacco species as outgroup revealed 10 out of 11 nodes with bootstrap values ≥ 95%, which is very high for an MP tree (Figure 9). The MP phylogenetic tree was even more strongly supported by eight 100% bootstrap values, showing that *Q. aquifolioides* was grouped with *Q. spinosa* within *Quercus*. Both of these are members of sect. *Heterobalanus*. The MP tree also revealed that *Q. rubra* and *Q. aliena* were the closest relatives of *Q. aquifolioides* and *Q. spinosa* (Figure 9). However, this phylogenetic tree is solely based on cp DNA. To fully understand their phylogenetic relationships, nuclear DNA is required to be investigated to assess the effect of introgression and hybridization on phylogeny.

## 3. Materials and Methods

### 3.1. Plant Material

We collected a *Q. aquifolioides* tree less than 3 years old from Lijiang Alpine Botanic Garden, China and transplanted it to Beijing Forestry University. *Q. aquifolioides* is a common, non-endangered tree species in China. No specific protective policy was implemented in this area. The plants were grown in a growth chamber under 150 mmol·m^−2^·s^−1^ light, with 16 h light/8 h dark cycles, at 24 °C with a constant humidity of 65%. Voucher specimens were deposited in the herbarium of Beijing Forestry University, Beijing, China.

### 3.2. Chloroplast Isolation, DNA Extraction, and Sequencing

A 0.3–0.5 g sample of the fresh young leaves was collected after the plant had grown in the dark for 24–36 h to promote starch degradation in chloroplasts (Nobel 1974). The chloroplast DNA (cpDNA) extraction and enrichment method followed the protocol developed by our group [50]. After amplifying the cpDNA by rolling circle amplification (RCA), we purified the RCA product and used 5 μg for library preparation. A 101-bp paired-end run was performed on an Illumina-HiSeq 1500 (Illumina, San Diego, CA, USA) at the gene sequencing platform of the School of Life Sciences, Tsinghua University, China. Briefly, library preparation was carried out following the manufacturer’s instructions with an insert size of up to 500 bp. Base calling was performed with RTA v.1.6 (Illumina, San Diego, CA, USA).

### 3.3. Chloroplast Genome Assembly

We assembled the *Q. aquifolioides* chloroplast genome using a pipeline developed in our lab [21]. Briefly, we used an in-house Perl script to eliminate low quality (probability of error > 1%) nucleotides in each read. SOAPdenovo 2 [51] was used for de novo assembly with default parameters, except that an insert size of 500 bp was set. Next, the primary contigs were assembled using the *Quercus rubra* chloroplast genome (GenBank accession number: JX970937) as the reference sequence. Gaps between two neighbor contigs were filled with N. These gaps were resolved as previously described [50].

### 3.4. Genome Annotation 

We used CpGAVAS [52] for chloroplast genome annotation then manually corrected the output. This program uses a chloroplast genome sequence in FASTA format to identify protein-coding genes by performing BLASTX searches against a custom database of known chloroplast genomes. The program also produces a circular map of the chloroplast genome, displaying the protein-coding genes, transfer RNAs (tRNAs), and ribosomal RNAs (rRNAs) based on the annotations. 

### 3.5. Repeat Analysis 

Simple sequence repeats (SSRs) in the cp genomes were detected using the Perl script MISA [53]. The thresholds set for the SSRs were 10, 6, 4, 3, 3, and 3 for mono-, di-, tri-, tetra-, penta-, and hexa-nucleotides, respectively. Tandem repeat sequences (>10 bp in length) were detected using the online program Tandem Repeats Finder [54]. The minimum alignment score and maximum period size were 90 and 500 respectively. The online REPuter software tool (Available online: https://bibiserv.cebitec.uni-bielefeld.de/reputer/) was used to identify forward, palindrome, reverse, and complement sequences with a minimum repeat size of 30 bp, and sequence identity greater than 90% (Hamming distance equal to 3) [55].

### 3.6. CCT Map

Comparative genome maps of *Q. aquifolioides* and the other three *Quercus* cp genomes were constructed by BLAST using CCT software [56] and the results were displayed as a circular map. Additional features such as the Clusters of Orthologous Groups of proteins (COG) and GC Skew in the reference genome were also included.

### 3.7. Sequence Divergence Analysis

The alignments of the cp genomes of *Q. aquifolioides* and the other three *Quercus* cp genome were visualized using mVISTA [57] (Available online: http://genome.lbl.gov/vista/mvista/submit.shtml) in Shuffle-LAGAN mode [34] in order to show interspecific variation. The sequence divergences of four *Quercus* protein coding genes were evaluated using MEGA 7 [58]. A sliding window analysis was conducted to generate nucleotide diversity (*Pi*) values for the three data sets (the aligned LSC, SSC, and IR regions of the four complete *Quercus* cp genomes) using DnaSP 5 [59]. The step size was set to 200 bp, with a 600 bp window length. The Tamura 3-parameter (T92) model was selected to calculate pairwise sequence divergences [60]. 

### 3.8. Selection Pressure Analysis

To estimate selection pressures, non-synonymous (Ka) and synonymous (Ks) substitution rates of 73 protein coding genes between the cp genomes of *Q. aquifolioides* and the other three *Quercus* species were calculated using DnaSP 5. For identification of site-specific selection, protein coding gene alignments were analyzed using Selecton [61], with *Q. aquifolioides* as a reference sequence. Two models, M8 (allows for positive selection operating on the protein) and M8a (does not allow for positive selection), were used and likelihood scores estimated by models were evaluated using a log-likelihood ratio test (LRT) with degree of freedom (df) = 1. Only sites with posterior probabilities > 0.8 were selected.

### 3.9. Phylogenetic Analysis

The sequences were aligned using MAFFT 7 [62]. Maximum parsimony (MP) analysis was executed using PAUP 4 [63]. A total of 73 protein-coding genes shared by all cp genomes were used for this phylogenetic analysis, which included 12 Fagaceae species (*Q. aquifolioides* KP340971; *Q. aliena* KP301144; *Q. rubra* JX970937; *Q. spinosa* KM841421; *Q. variabilis* KU240009; *Q. dolicholepis* KU240010; *Q. baronii* KT963087; *Castanopsis concinna* KT793041; *C. echinocarpa* KJ001129; *Castanea henryi* KX954615; *Lithocarpus balansae* KP299291; *Trigonobalanus doichangensis* KF990556), with two *Nicotiana* species (*N. sylvestris* AB237912; *N. tabacum* Z00044) as outgroups. 

## Figures and Tables

**Table 1:** Summary of the features of four complete *Quercus* plastomes.

Genome Features	Sect. *Heterobalanus*		Sect. *Lobatae*	Sect. *Quercus*
	*Q. aquifolioides*	*Q. spinosa*	*Q. rubra*	*Q. aliena*
Genome size/GC content	160,415/37.0	160,825/36.9	161,304/36.8	160,921/36.9
Coding genes: number/size	80(7)/80,270	80(7)/80,812	80(7)/80,946	80(7)/80,052
tRNA: number/size	39/10,625	38/11,402	39/10,756	39/10,753
rRNA: number/size	8/9048	8/9050	8/9050	8/9048
LSC: size/percent/GC content	89,807/56/34.8	90,371/56.2/34.7	90,541/56.1/34.7	90,258/56.1/34.7
SSC: size/percent/GC content	18,894/11.8/31.2	18,732/11.6/31.2	19,023/56.1/30.9	18,972/11.8/31.0
IR: size/percent/GC content	51,754/32.2/42.7	51,722/32.2/47.2	52,740/32.7/42.7	51,682/32.1/42.7
Introns: size/percent	20,473/12.8	19,757/12.3	20,217/12.5	20,014/12.4
Intergenic spacer: size/percent	49,548/31.0	50,207/31.2	47,473/29.4	47,304/29.3

Numbers in brackets denote the numbers of genes duplicated in the IR regions.

**Table 2:** Positive selection sites identified by Selecton.

Gene	NULL (M8a)	POSITIVE (M8)	Putative Sites under Positive Selection
*rpl2*	−1177.05	−1174.54	1 (131 S)
*ycf2*	−9154.8	−9154.87	5 (96 K, 932 W, 1174 P, 1291 W, 2007 R)
*rps7*	−615.83	−615.616	1 (130 E)
*ndhD*	−2145.31	−2141.84	8 (170 T, 188 G, 200 L, 206 A, 362 R, 375 P, 413 Q, 504 F)
*ycf1*	−8057.78	−8051.12	8 (426 F, 529 L, 757 L, 761 L, 1007 I, 1490 Q, 1491 G, 1492 F)
*rpoC2*	−5814.68	−5814.8	26 (33 H, 131 P, 280 L, 364 I, 505 H, 542 E, 587 E, 595 P, 598 V, 626 N, 643 K, 691 G, 697 T, 815 Y, 849 G, 856 H, 898 D, 947 S, 1013 K, 1074 I, 1081 A, 1132 E, 1176 I, 1273 C, 1374 D, 1394 N)
*rpoC1*	−2857.37	−2855.15	1 (145 Y)
*psaB*	−2999.97	−2998.62	3 (145 L, 238 E, 239 K)
*ndhJ*	−633.793	−633.569	1 (107 A)
*ndhC*	−828.789	−828.761	2 (68 V, 86 F)
*rpl36*	−145.376	−145.135	1 (20 R)

Likelihood ratio test (LRT) analysis of models comparison M8 vs. M8a. M8 represents a model with positive selection; M8a represents null model without positive selection. Degree of freedom (df) = 1.

## Supplementary Materials

Supplementary materials can be found at http://www.mdpi.com/1422-0067/19/4/1042/s1.
